# Supplementary figures and images for: Inhibitory Effects of Bovine Lactoferricin-Lactoferrampin on Senecavirus A and Foot-and-Mouth Disease Virus with Recombinant Lactobacillus Oral Treatment in Mice
Source: Vet Sci. 2025 Feb 25;12(3):199. doi: 10.3390/vetsci12030199 (PMC11945493; doi:10.3390/vetsci12030199)

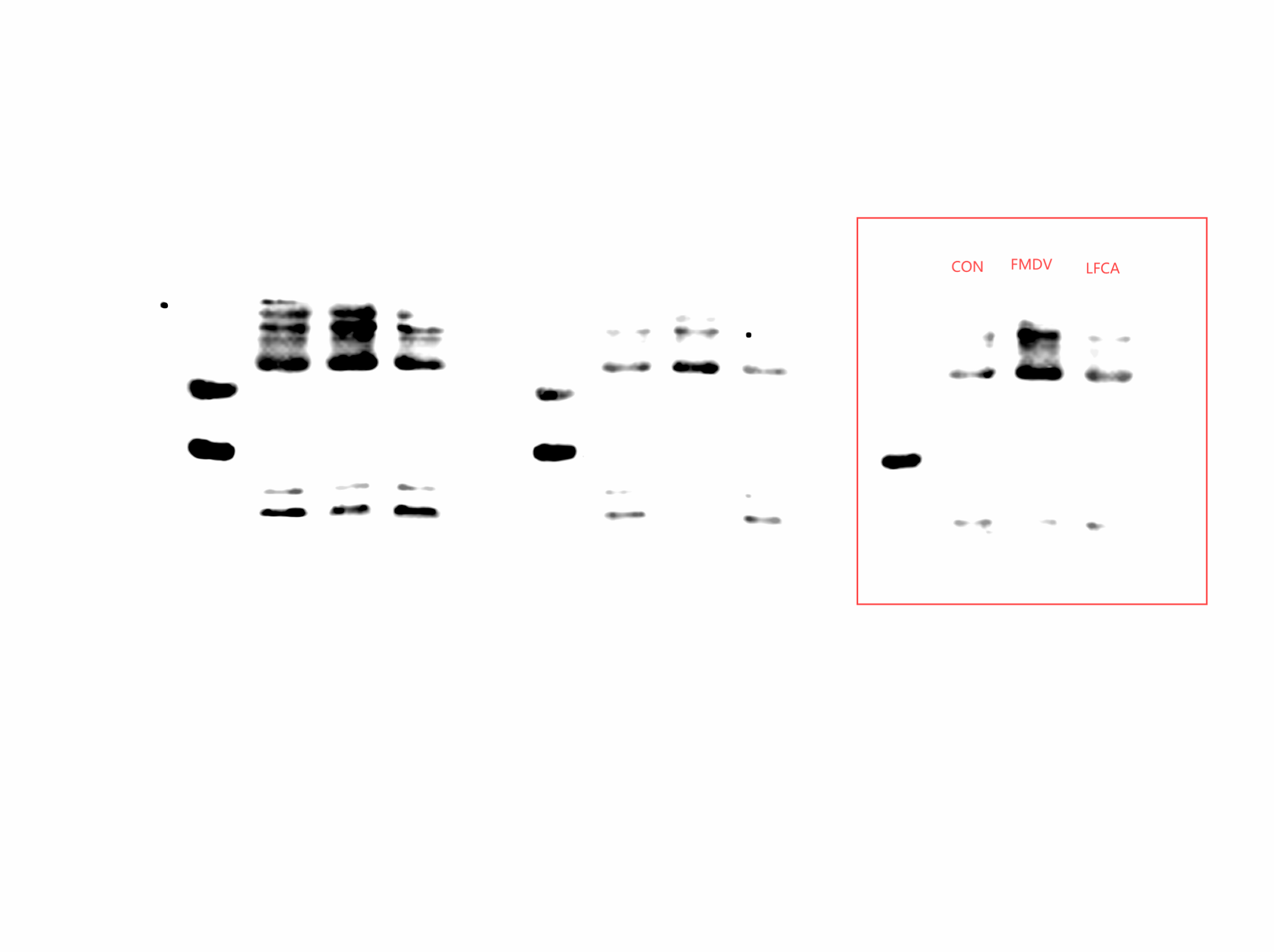

Supplement: Supplementary file 1 [file vetsci-12-00199-s001.zip › vetsci-3448808-supplementary/Original Images/Figure 5b.FMDV-ho1.png]

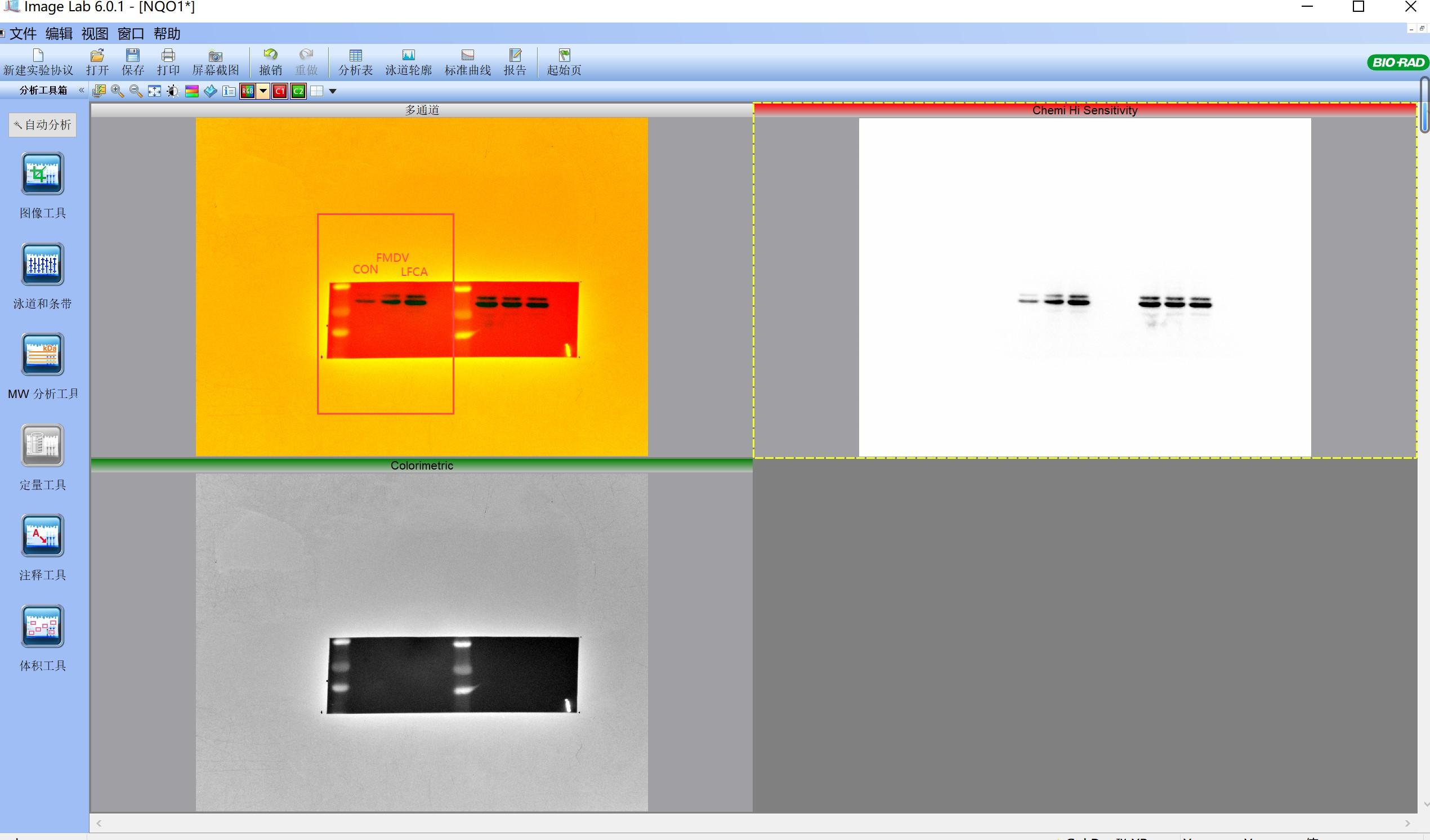

Supplement: Supplementary file 1 [file vetsci-12-00199-s001.zip › vetsci-3448808-supplementary/Original Images/Figure 5b.FMDV-NQO1.jpg]

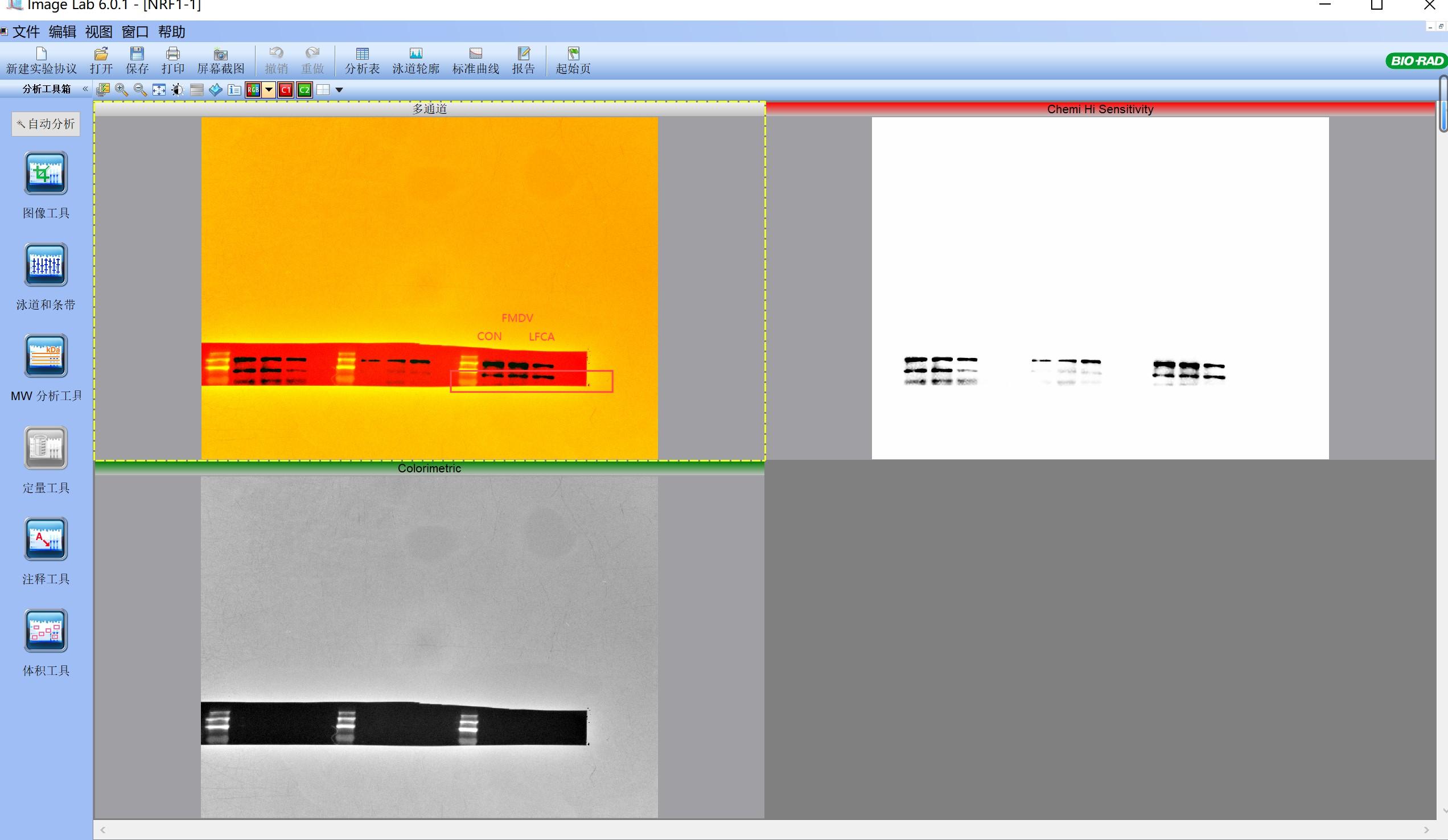

Supplement: Supplementary file 1 [file vetsci-12-00199-s001.zip › vetsci-3448808-supplementary/Original Images/Figure 5b.FMDV-NRF2.jpg]

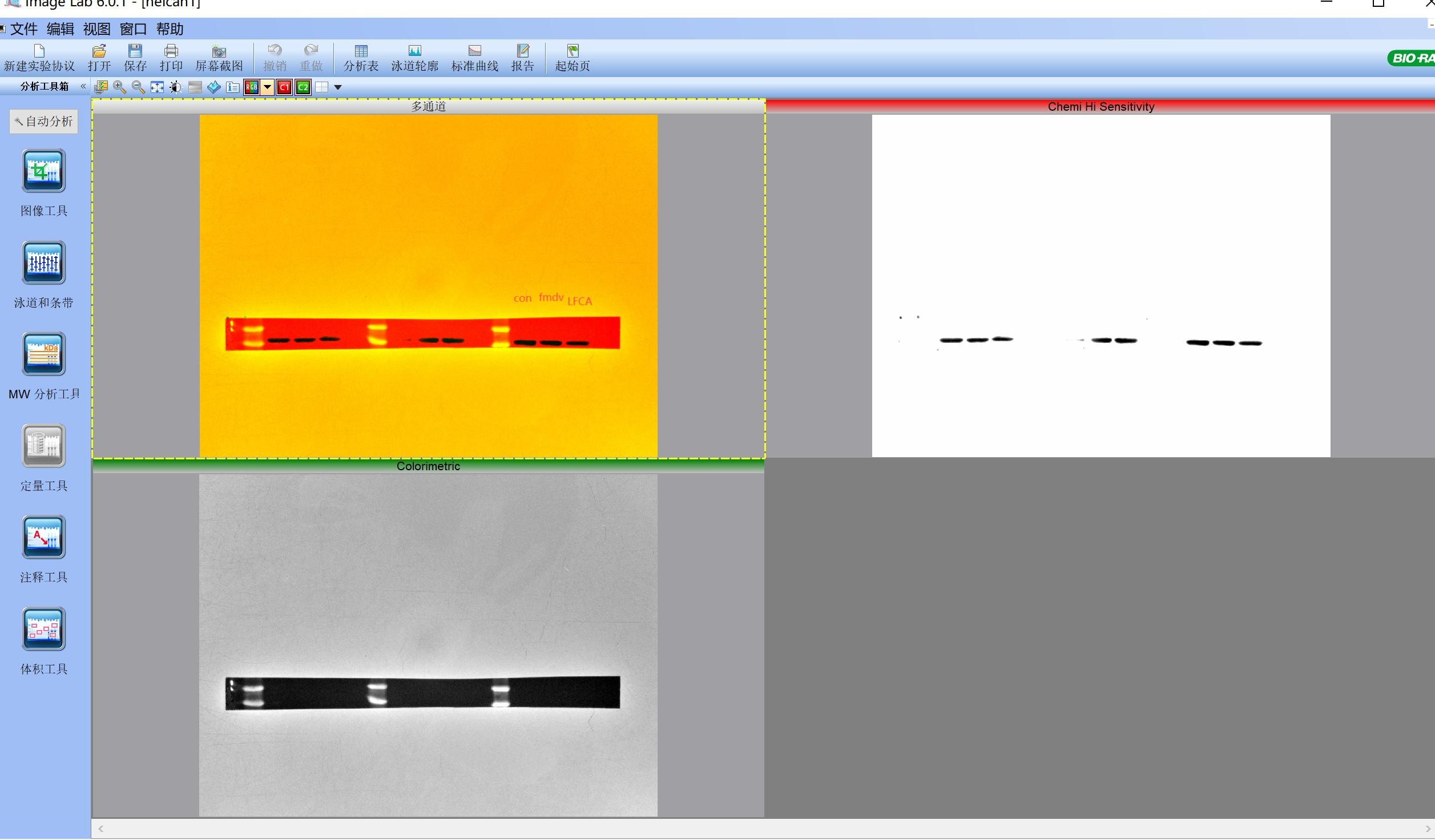

Supplement: Supplementary file 1 [file vetsci-12-00199-s001.zip › vetsci-3448808-supplementary/Original Images/Figure 5b.FMDV-β-tublin.jpg]

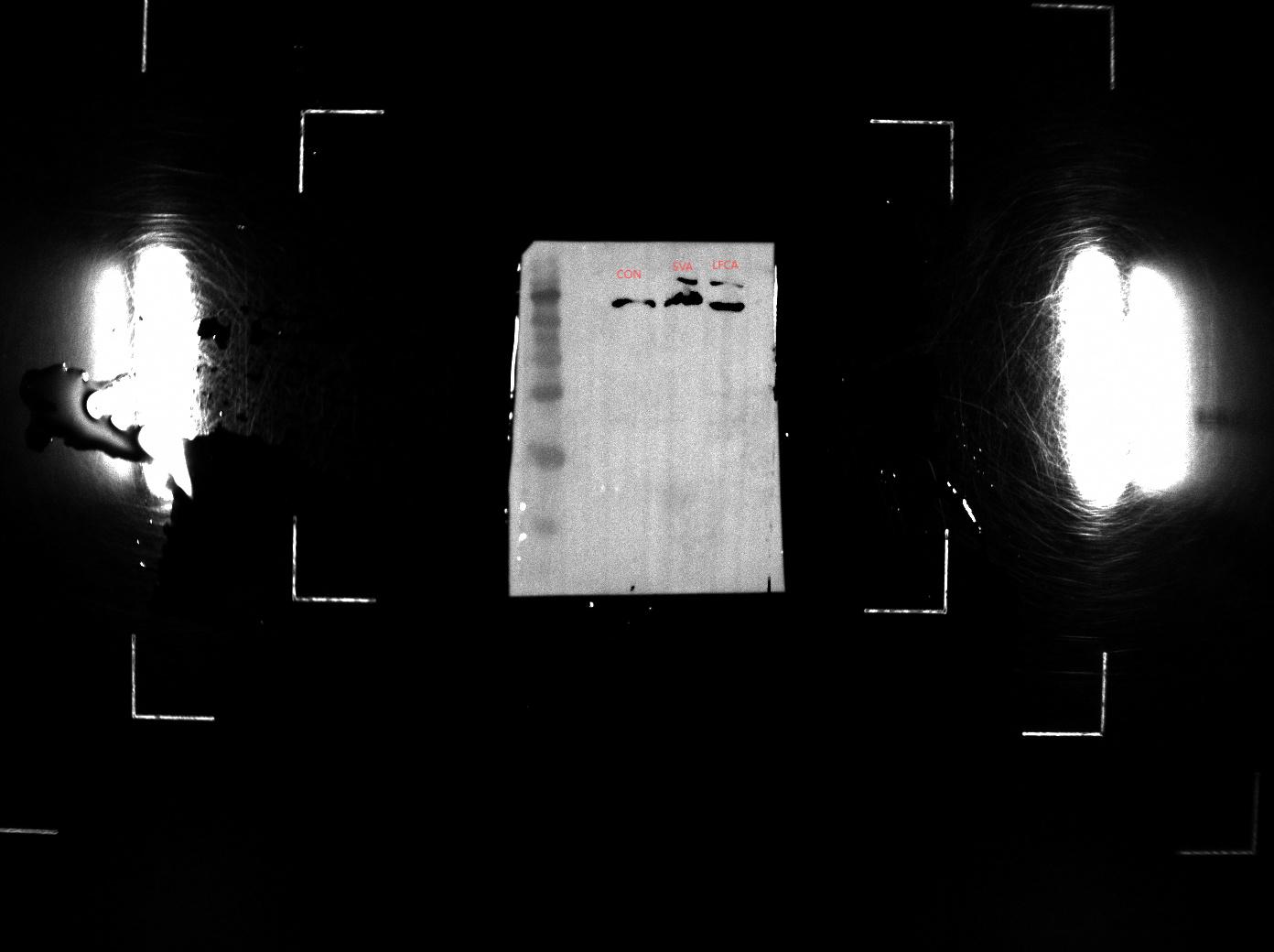

Supplement: Supplementary file 1 [file vetsci-12-00199-s001.zip › vetsci-3448808-supplementary/Original Images/Figure 5b.SVA-HO-1.jpg]

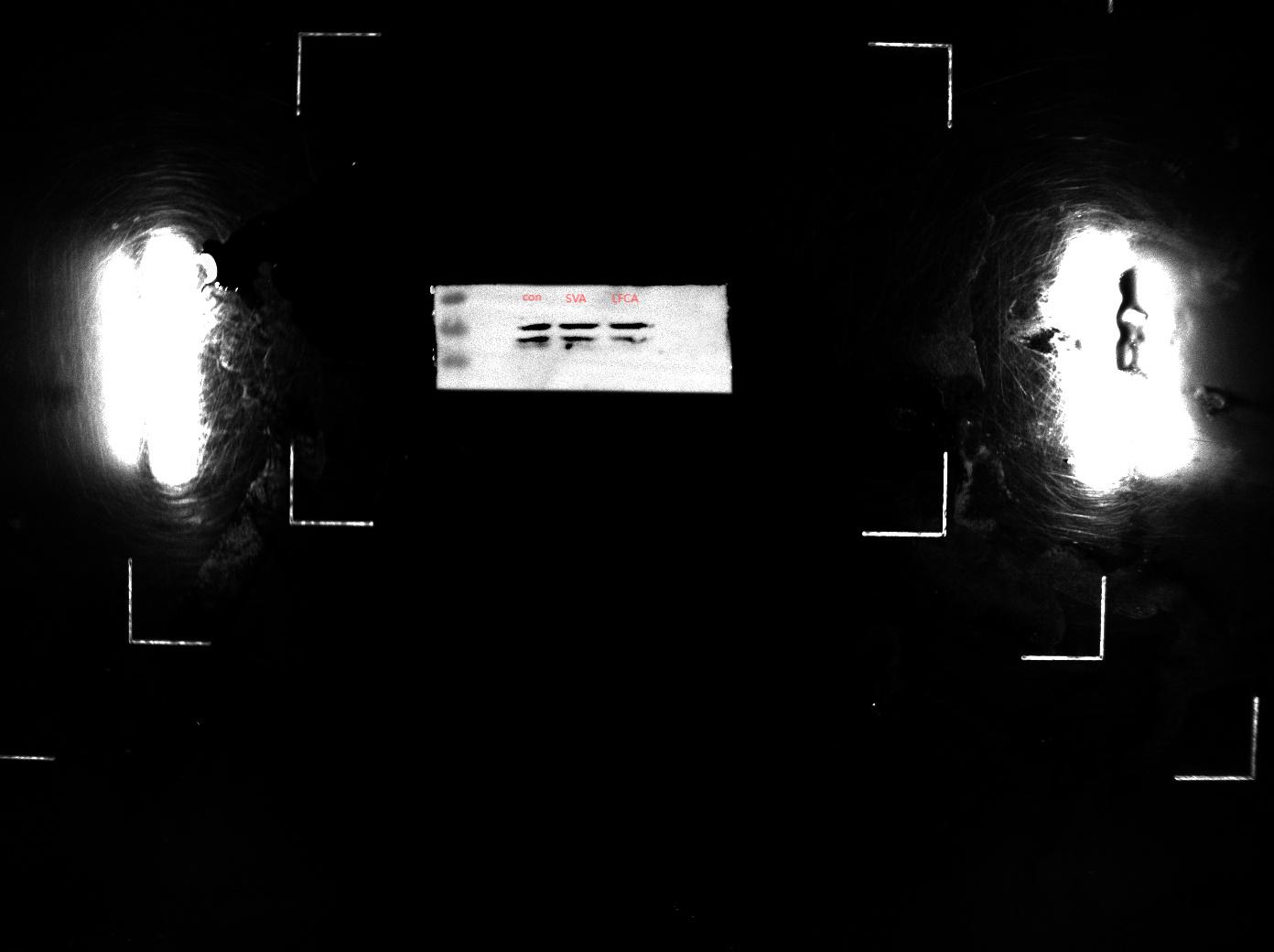

Supplement: Supplementary file 1 [file vetsci-12-00199-s001.zip › vetsci-3448808-supplementary/Original Images/Figure 5b.SVA-NQO1.jpg]

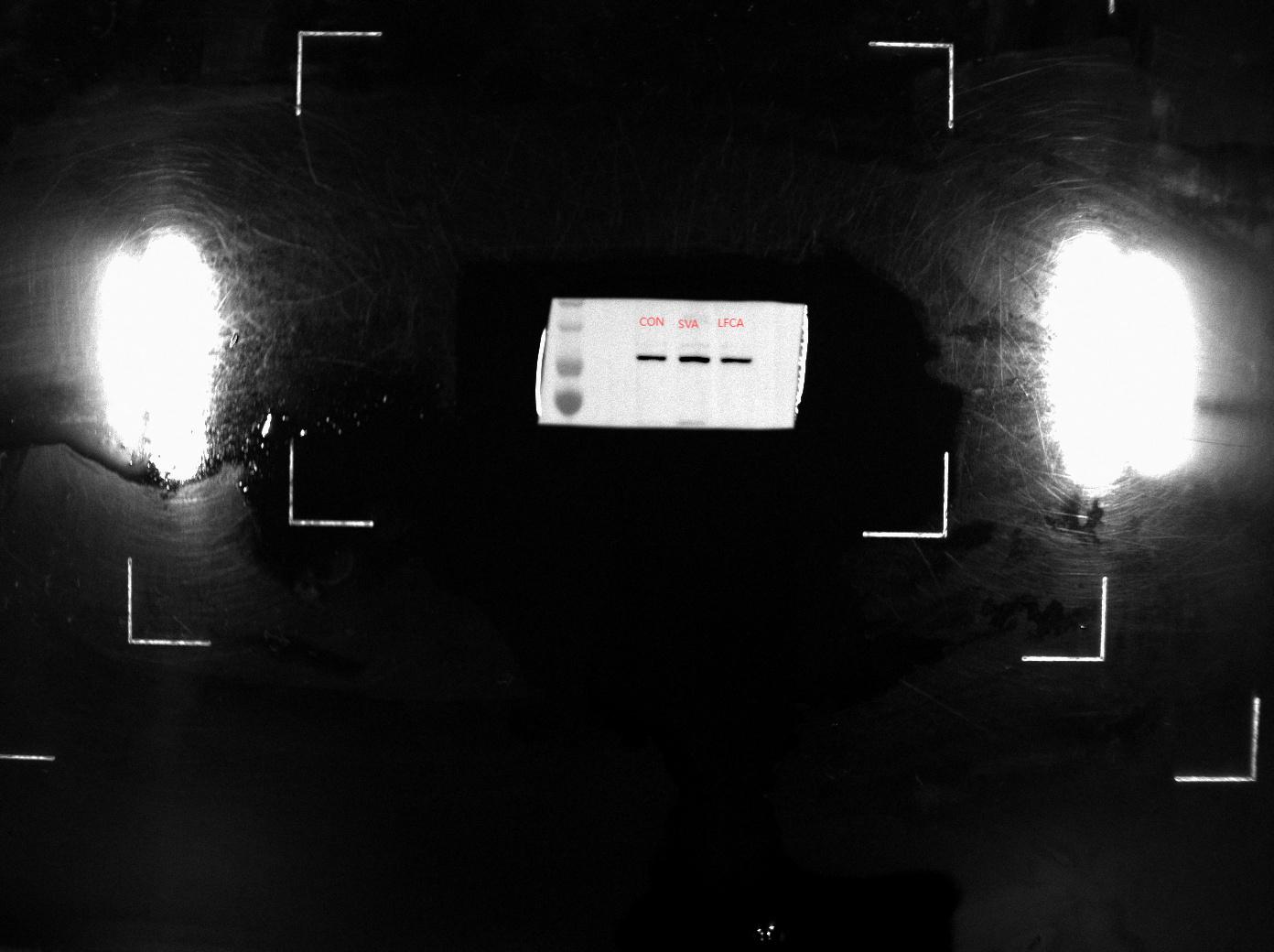

Supplement: Supplementary file 1 [file vetsci-12-00199-s001.zip › vetsci-3448808-supplementary/Original Images/Figure 5b.SVA-NRF2.jpg]

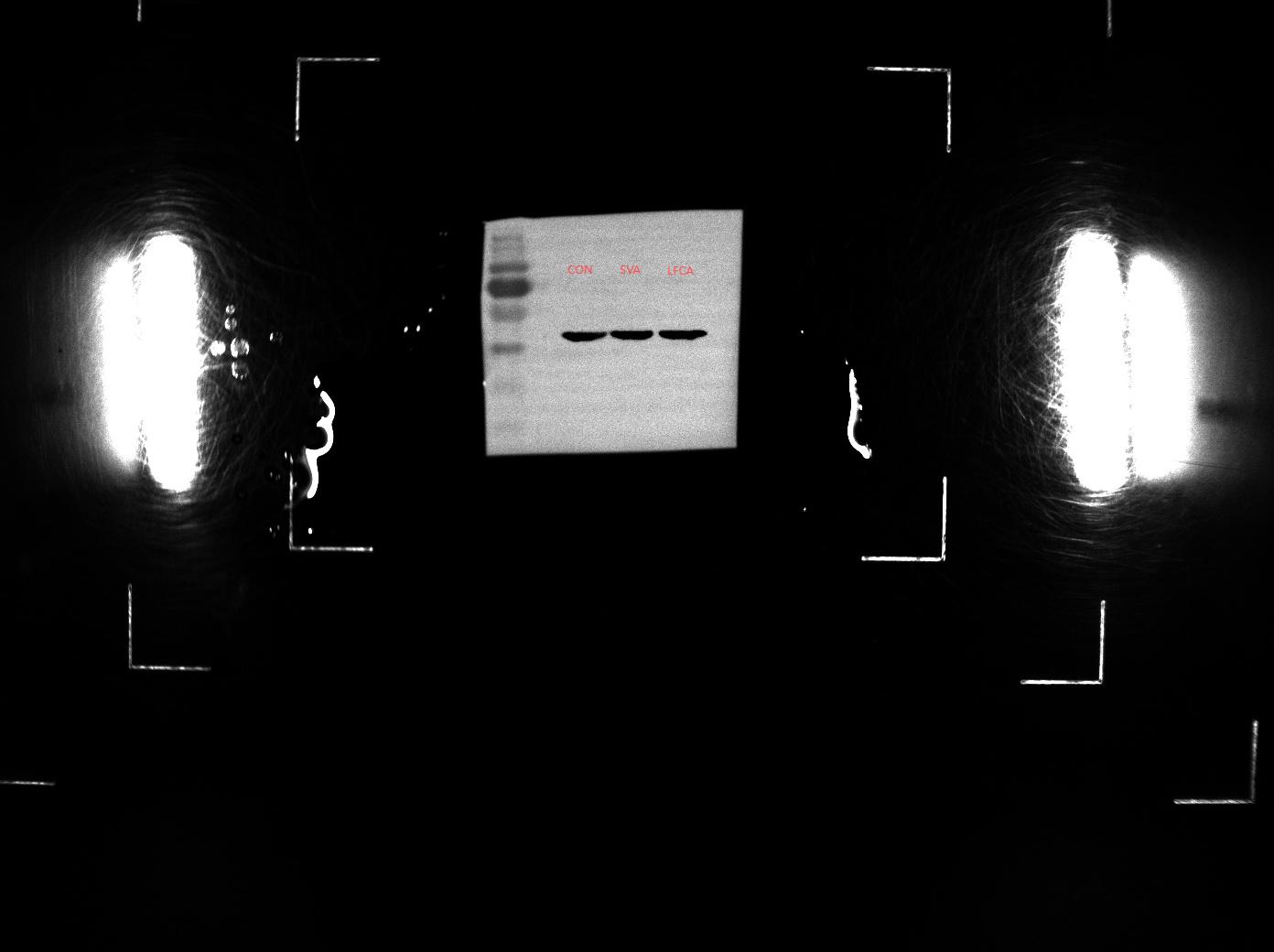

Supplement: Supplementary file 1 [file vetsci-12-00199-s001.zip › vetsci-3448808-supplementary/Original Images/Figure 5b.SVA-β-actin.jpg]

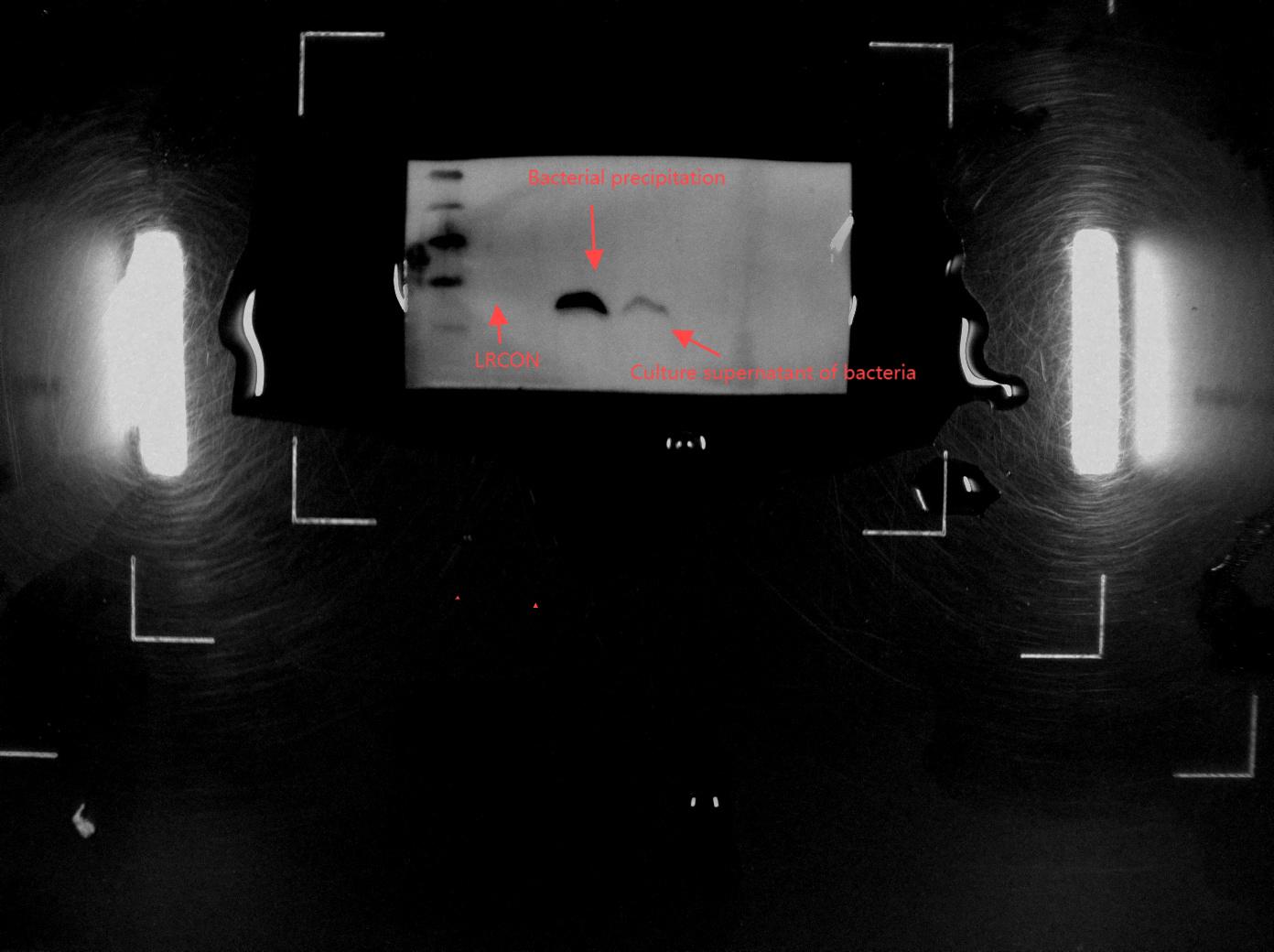

Supplement: Supplementary file 1 [file vetsci-12-00199-s001.zip › vetsci-3448808-supplementary/Original Images/Figure 6b-ii..jpg]

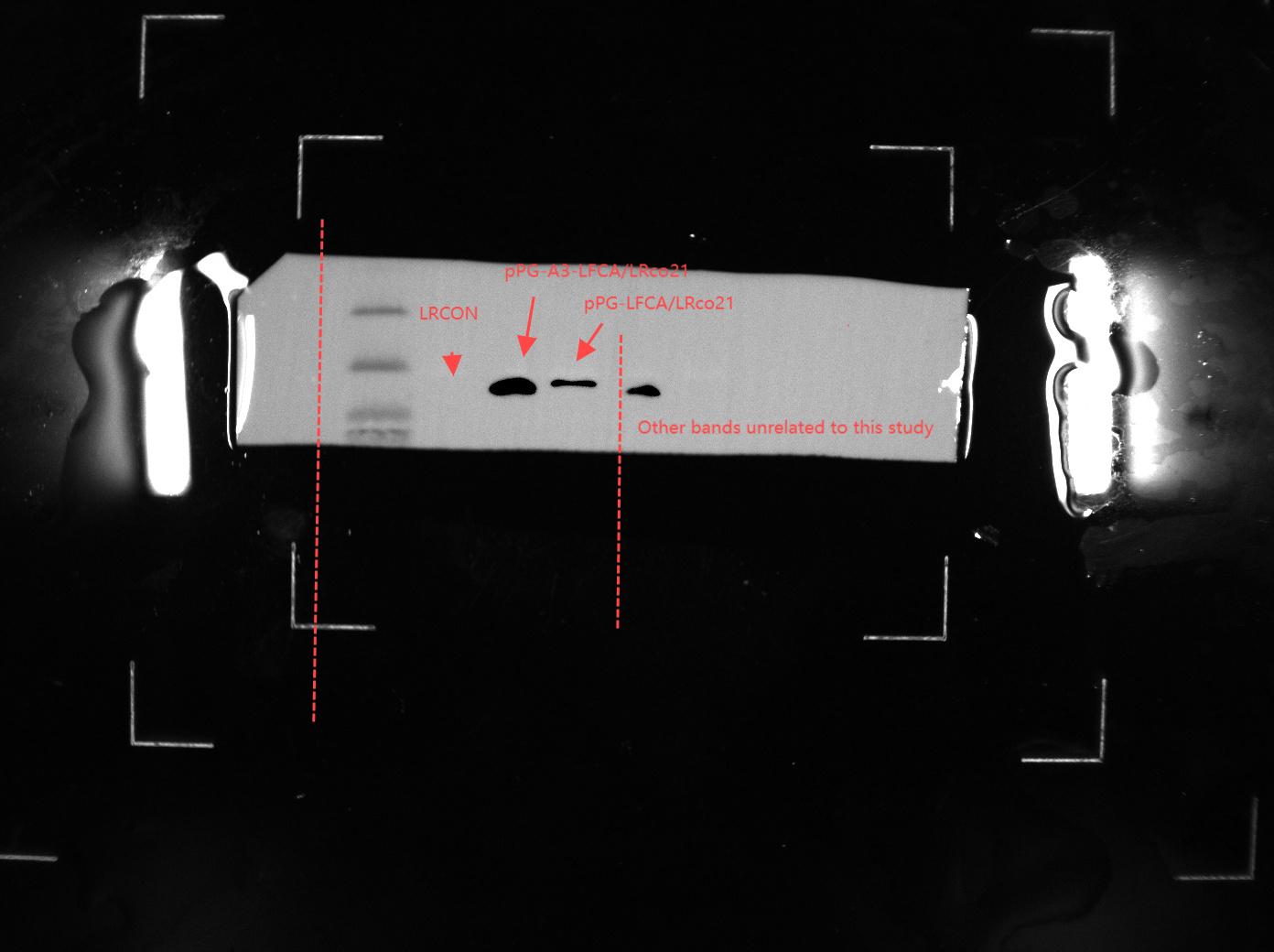

Supplement: Supplementary file 1 [file vetsci-12-00199-s001.zip › vetsci-3448808-supplementary/Original Images/Figure 6bi..jpg]

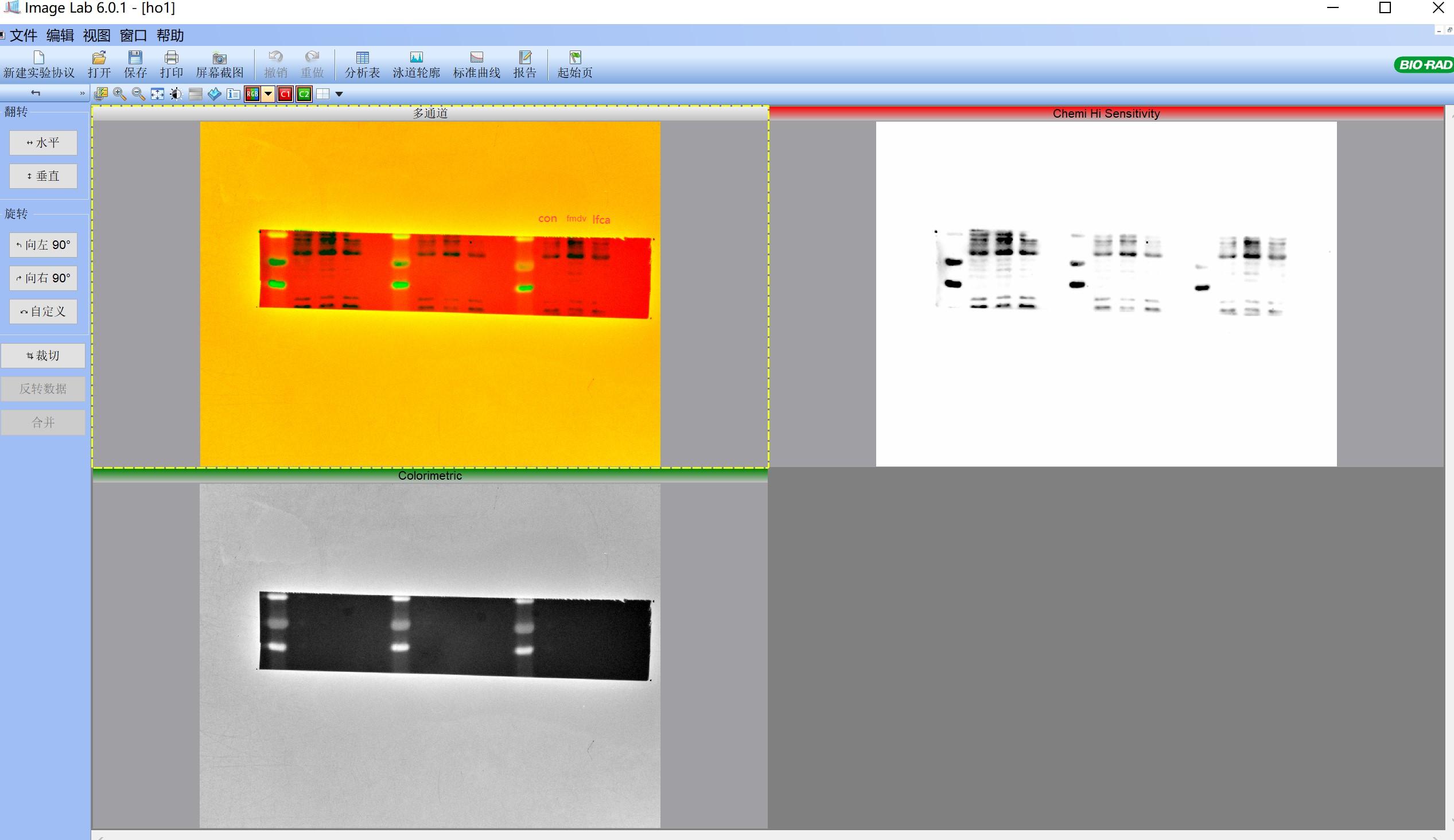

Supplement: Supplementary file 1 [file vetsci-12-00199-s001.zip › vetsci-3448808-supplementary/Original Images/Figure S5b.FMDV-HO-1.jpg]
